# Triglyceride-Glucose Index and Risk of End-Stage Kidney Disease in Young Adults: National Population-Based Cohort Study

**DOI:** 10.2196/73750

**Published:** 2026-06-03

**Authors:** Jin Sug Kim, Yu Ho Lee, Sungjin Chung, Hyeon Seok Hwang, Kyungdo Han, Hye Eun Yoon

**Affiliations:** 1 Division of Nephrology, Department of Internal Medicine, Kyung Hee University College of Medicine, Kyung Hee University Medical Center, Seoul, Republic of Korea; 2 Division of Nephrology, Department of Internal Medicine, Yeouido St. Mary's Hospital, College of Medicine, The Catholic University of Korea, Seoul, Republic of Korea; 3 Department of Statistics and Actuarial Science, Soongsil University, Seoul, Republic of Korea; 4 Division of Nephrology, Department of Internal Medicine, Seoul St. Mary’s Hospital, College of Medicine, The Catholic University of Korea, 222 Banpo-daero, Seocho-gu, Seoul, 06591, Republic of Korea, 82 2 2258 6854; 5 Transplantation Research Center, College of Medicine, The Catholic University of Korea, Seoul, Republic of Korea

**Keywords:** triglyceride-glucose index, chronic kidney disease, end-stage kidney disease, dialysis, young adults

## Abstract

**Background:**

The triglyceride-glucose (TyG) index, a simple marker of insulin resistance, is associated with chronic kidney disease and end-stage kidney disease (ESKD). However, its association with ESKD in young adults remains unexplored.

**Objective:**

This study aimed to investigate whether the TyG index increases the risk of ESKD in the young adult population.

**Methods:**

This nationwide retrospective, population-based cohort study analyzed data from 6,555,863 individuals aged 20‐39 years between January 2009 and December 2012 using the Korean National Health Insurance Database. Participants were categorized into quartiles and deciles based on the TyG index. The cumulative incidence and adjusted hazard ratios for ESKD were assessed.

**Results:**

Over a median follow-up period of 10.61 years, 4910 (0.75%) participants developed ESKD. The incidence of ESKD increased with a higher TyG index in quartiles and deciles. The adjusted hazard ratio for ESKD in the highest quartile was 3.438 (95% CI 3.096‐3.818) compared to the lowest quartile, and that in the highest decile was 5.944 (95% CI 5.022‐7.035) compared to the lowest decile. These associations remained consistent across subgroup analyses by age, sex, obesity, dyslipidemia, and chronic kidney disease.

**Conclusions:**

The TyG index is significantly associated with ESKD risk in young adults.

## Introduction

Chronic kidney disease (CKD) is a significant global health issue and a leading cause of mortality and morbidity. Its prevalence is rapidly increasing, with approximately 843.6 million patients affected globally in 2017 [1]. Additionally, CKD and its associated comorbidities impose a significant financial burden on the health care systems [2]. Although CKD is predominantly diagnosed in older adults, its global incidence among adolescents and young adults is increasing [3]. This trend is attributed to the increasing rates of obesity, lipid abnormalities, diabetes, and hypertension in childhood or adolescence [3-5]. Consequently, young adults are more susceptible to developing CKD than in the past. Moreover, early onset CKD is associated with greater disease severity and cardiovascular complications because of prolonged disease duration [6], as well as low quality of life and socioeconomic burden [7].

Insulin resistance is an early metabolic alteration in CKD [8], and its association with CKD is independent of diabetes [9,10]. It contributes to progressive kidney damage by causing glomerular hyperfiltration, sodium retention, tubular dysfunction, and kidney inflammation and fibrosis [11]. Although the hyperinsulinemic-euglycemic glucose clamp technique is considered the gold standard for measuring insulin resistance, its clinical utility is limited because of the time and resources required. The homeostatic model assessment of insulin resistance is a more commonly used method [8] and has been reported to increase the risk of incident CKD development in individuals with normal kidney function [12]. The triglyceride-glucose (TyG) index, calculated as the logarithmic product of fasting triglyceride and glucose levels, correlates well with both the hyperinsulinemic-euglycemic clamp and homeostatic model assessment of insulin resistance indices [13]. Owing to its simplicity and reliability, the TyG index is widely used in large-scale epidemiological studies to assess insulin resistance.

Previous studies have established an association between the TyG index and incident CKD in the general population [14]. Recently, an association has also been reported between the TyG index and end-stage kidney disease (ESKD) in the general population [15] and in patients with diabetes [16]. However, no studies have examined the relationship between the TyG index and the development of ESKD, specifically in young adults. Therefore, this study aimed to investigate the association between the TyG index and the risk of incident ESKD in this population.

## Methods

### Database

This study retrospectively analyzed a nationwide cohort of patients who underwent health checkups between 2009 and 2012, as provided by the National Health Insurance Service (NHIS) of South Korea. The NHIS database includes individual health checkup results, along with claims data containing diagnostic, procedural, and prescription codes. As the NHIS serves as the sole public insurer in South Korea, its database is representative of the Korean population. The health checkup database comprises demographic data, including body measurements, blood and urine test results, and survey results on health-related behaviors.

### Study Design and Population

This study identified 6,891,401 individuals aged 20‐39 years who underwent a national health checkup between 2009 and 2012. We excluded 330,334 individuals with insufficient data to calculate the TyG index; 2412 individuals with ESKD identified using rare incurable disease registry codes for hemodialysis (V001), peritoneal dialysis (V003), or kidney transplantation (V005); and 2732 individuals with insurance claims related to ESKD or who died within 1 year after the index date. The remaining 6,555,863 eligible individuals were included and followed up until December 2021. Regarding the time frame, the start of follow-up (time zero) was defined as 1 year after the index date (lag period). Follow-up was stopped at death, censorship (eg, immigration), or ESKD development. The mean follow-up duration was 10.34 (SD 1.18) years. The participants were divided into 4 groups based on their TyG index, with quartiles 1 and 4 representing the lowest and the highest values, respectively. The mean (SD) values of the TyG index for each quartile were as follows: quartile 1: 7.68 (SD 0.29), quartile 2: 8.16 (SD 0.27), quartile 3: 8.52 (SD 0.32), and quartile 4: 9.15 (SD 0.51).

### Clinical and Laboratory Measurements

All participants completed a questionnaire detailing their medical history and lifestyle habits, including smoking, alcohol consumption, and exercise. Smoking status was categorized as nonsmoker, former smoker, or current smoker. Alcohol consumption was classified as nondrinker, mild drinker (<30 g/day), or heavy drinker (≥30 g/day). Regular exercise was defined as vigorous-intensity exercise performed 3 or more times per week or moderate-intensity exercise performed 5 or more times per week. Routine physical examinations included body weight, height, waist circumference, and blood pressure. BMI was calculated as body weight (kg) divided by height squared (m^2^). A low income was defined as being in the lowest quartile of the NHIS population or receiving government-funded medical aid. Blood samples were collected after overnight fasting to assess the serum levels of glucose, creatinine, total cholesterol, triglycerides, high-density lipoprotein-cholesterol (HDL-C), and low-density lipoprotein-cholesterol. The estimated glomerular filtration rate (eGFR) was calculated using the Modification of Diet in Renal Disease equation [17].

### Definitions of Variables and Study Outcome

The TyG index was calculated using the formula log (fasting triglyceride [mg/dL] × fasting blood glucose [mg/dL]/2) [13,18]. Baseline comorbidities were defined as follows: hypertension (*ICD-10* [*International Statistical Classification of Diseases and Related Health Problems, 10th Revision*] codes I10-I13 or I15 and treatment with antihypertensive medications, systolic blood pressure [SBP] ≥ 140 mmHg, or diastolic blood pressure [DBP] ≥ 90 mmHg), diabetes (*ICD-10* codes E11-E14 and antidiabetic treatment or fasting glucose level ≥ 126 mg/dL), dyslipidemia (*ICD-10* code E78 with lipid-lowering agents or serum total cholesterol ≥240 mg/dL), and CKD (eGFR<60 mL/min/1.73 m^2^). Antihypertensive medications included angiotensin type II receptor blockers, angiotensin-converting enzyme inhibitors, calcium channel blockers, beta-blockers, diuretics, or alpha-blockers. Antidiabetic medications included metformin, meglitinide, sulfonylurea, dipeptidyl peptidase IV inhibitor, alpha-glucosidase inhibitor, thiazolidines, or insulin. Lipid-lowering agents (dyslipidemia medication) included statins, ezetimibe, or fibrates.

In this study, obesity was defined as a BMI ≥25 kg/m^2^, consistent with Asian-specific criteria [19]. Metabolic syndrome (MetS) was diagnosed based on the revised National Cholesterol Education Program Adult Treatment Panel III criteria [20,21]. MetS was diagnosed when three or more of the following five criteria were met: (1) elevated blood pressure (SBP ≥130 mmHg, DBP ≥85 mmHg, use of antihypertensive medications at baseline, and/or a history of hypertension), (2) elevated triglycerides (fasting triglycerides  ≥ 150 mg/dL or use of dyslipidemia medications at baseline), (3) reduced HDL-C (<40 mg/dL for men and <50 mg/dL for women), (4) elevated fasting plasma glucose level (≥100 mg/dL or use of medications for elevated glucose levels), and (5) abdominal obesity (waist circumference ≥90 cm for men or ≥85 cm for women).

The primary outcome of this study was newly diagnosed ESKD, defined as the initiation of kidney replacement therapy and/or kidney transplantation. Kidney replacement therapy or kidney transplantation was defined based on the rare incurable disease registry codes for hemodialysis (V001), peritoneal dialysis (V003), or kidney transplantation (V005).

### Statistics Analysis

Categorical variables are presented as frequencies and percentages, whereas continuous variables are expressed as mean (SD). For continuous variables not following a normal distribution, log transformation was applied, and the results were presented as geometric means with 95% CIs. Baseline characteristics across the TyG index quartiles were compared using one-way ANOVA for continuous variables and the chi-square test for categorical variables. The follow-up duration was calculated for each TyG index quartile group. The incidence rates of ESKD were determined for the TyG index, fasting glucose, and triglyceride quartiles over the total follow-up period for the study outcome. Incidence rates were expressed as events per 1000 person-years. Kaplan-Meier curves were generated to estimate incidence trends, and the log-rank test was used to compare groups. All outcomes were analyzed using Cox proportional hazards regression analysis while controlling for baseline covariates. Sensitivity analyses were performed to reduce potential bias and demonstrate the consistency of our findings. Incidence rates of ESKD were calculated for deciles of the TyG index, fasting glucose, and triglycerides. Subgroup analyses were performed to explore whether the association between the TyG index and ESKD differed by age, sex, obesity, MetS, diabetes, diabetes medication, dyslipidemia, dyslipidemia medication, and CKD using Cox proportional hazard regression analysis adjusted for age, sex, BMI, smoking, drinking, regular exercise, low income, diabetes medication, dyslipidemia medication, hypertension, eGFR, and CKD. Additionally, 2-year follow-up values of the TyG index, fasting glucose, and triglyceride were used to analyze the incidence rates of ESKD and the association between the TyG index quartiles and ESKD. The incidence rates and associations were evaluated both in terms of the 2-year follow-up values and the time-averaged values of baseline and 2-year follow-up. The risks of ESKD associated with the TyG index groups were expressed as hazard ratios (HRs) with 95% CIs, using the first quartile or decile group as the reference. A two-tailed *P*<.05 was considered statistically significant. All analyses were performed using SAS (version 9.4; SAS Institute) and R (version 3.4.1; R Foundation for Statistical Computing).

### Ethical Considerations

This study adhered to the Declaration of Helsinki (2013 revision) and was approved by the Institutional Review Board of CHA Bundang Medical Center (CHAMC 2024-04-034). The requirement for written informed consent was waived by the Institutional Review Board because of the retrospective nature of the study.

## Results

### Baseline Characteristics

Baseline demographic and clinical characteristics were compared across the TyG index quartiles (Table 1). An increase in the TyG index quartile was associated with significant increases in age, BMI, waist circumference, SBP, DBP, and levels of fasting plasma glucose, total cholesterol, and triglycerides, whereas HDL-C and eGFR levels decreased. Similarly, higher TyG index quartiles were associated with significantly greater proportions of individuals with high BMI, current smoking status, heavy alcohol consumption, and use of diabetes or dyslipidemia medication. The proportion of individuals with low income varied across the TyG index quartiles, with lower prevalence in the middle quartiles. Additionally, the prevalence of hypertension, diabetes, and dyslipidemia increased across quartiles, whereas the proportion of individuals engaging in regular exercise decreased. Elevated low-density lipoprotein-cholesterol levels and a higher prevalence of CKD were also observed in the higher TyG index quartiles.

**Table 1.** Baseline characteristics of quartiles of the triglyceride-glucose index.

Variable	Quartile 1 (n= 1,638,852)	Quartile 2 (n= 1,639,754)	Quartile 3 (n= 1,637,725)	Quartile 4 (n= 1,639,532)	*P* value
TyG^a^ index, mean (SD)	7.68 (0.29)	8.16 (0.27)	8.52 (0.32)	9.15 (0.51)	—^b^
Age (years), mean (SD)	29.47 (4.92)	30.5 (4.94)	31.26 (4.91)	32.24 (4.75)	<.001
Sex, n (%)					.35
Male	975,747 (59.54)	975,000 (59.46)	975,117 (59.54)	975,291 (59.49)	
Female	663,105 (40.46)	664,754 (40.54)	662,608 (40.46)	664,241 (40.51)	
BMI (kg/m^2^), mean (SD)	21.65 (2.86)	22.38 (3.22)	23.24 (3.56)	24.74 (3.94)	<.001
BMI category, n (%)					<.001
<18.5	182,525 (11.14)	144,074 (8.79)	109,906 (6.71)	59,424 (3.62)	
18.5≤ BMI<23	983,454 (60.01)	859,700 (52.43)	716,050 (43.72)	502,144 (30.63)	
23≤ BMI<25	273,285 (16.68)	315,712 (19.25)	338,387 (20.66)	333,491 (20.34)	
25≤ BMI<30	183,066 (11.17)	284,045 (17.32)	403,750 (24.65)	588,208 (35.88)	
≥30	16,522 (1.01)	36,223 (2.21)	69,632 (4.25)	156,265 (9.53)	
Smoking, n (%)					<.001
Non	981,155 (59.87)	925,478 (56.44)	877,695 (53.59)	806,929 (49.22)	
Former	158,638 (9.68)	169,509 (10.34)	175,628 (10.72)	175,936 (10.73)	
Current	499,059 (30.45)	544,767 (33.22)	584,402 (35.68)	656,667 (40.05)	
Drinking, n (%)					<.001
Non	650,144 (39.67)	627,375 (38.26)	608,019 (37.13)	586,979 (35.8)	
Mild	884,508 (53.97)	887,051 (54.1)	880,779 (53.78)	852,384 (51.99)	
Heavy	104,200 (6.36)	125,328 (7.64)	148,927 (9.09)	200,169 (12.21)	
Regular exercise, n (%)	244,252 (14.9)	212,375 (12.95)	198,244 (12.1)	187,284 (11.42)	<.001
Low income, n (%)	363,926 (22.21)	342,111 (20.86)	335,569 (20.49)	352,120 (21.48)	<.001
Hypertension, n (%)	57,896 (3.53)	86,336 (5.27)	124,874 (7.62)	212,860 (12.98)	<.001
Diabetes, n (%)	3085 (0.19)	7909 (0.48)	17,994 (1.1)	98,135 (5.99)	<.001
Dyslipidemia, n (%)	25,780 (1.57)	57,167 (3.49)	109,247 (6.67)	254,963 (15.55)	<.001
Diabetes medication, n (%)	1496 (0.09)	2292 (0.14)	4368 (0.27)	20,832 (1.27)	<.001
Dyslipidemia medication, n (%)	3686 (0.22)	7218 (0.44)	13,155 (0.8)	33,633 (2.05)	<.001
CKD^c^, n (%)	43,725 (2.67)	41,914 (2.56)	42,859 (2.62)	47,590 (2.9)	<.001
Waist circumference (cm), mean (SD)	74.12 (8.39)	76.06 (9.29)	78.17 (10.03)	81.75 (10.55)	<.001
SBP^d^, mmHg, mean (SD)	114.99 (12.13)	116.52 (12.58)	118.2 (13.1)	121.21 (14.03)	<.001
DBP^e^, mmHg, mean (SD)	71.71 (8.68)	72.92 (9)	74.16 (9.38)	76.32 (10.07)	<.001
Fasting glucose (mg/dL), mean (SD)	85.07 (9.47)	88.62 (10.14)	91.16 (11.44)	98.71 (26.15)	<.001
Total cholesterol (mg/dL), mean (SD)	169.55 (27.9)	179.54 (29.72)	187.9 (31.95)	201.35 (36.74)	<.001
HDL-C^f^ (mg/dL), mean (SD)	61.3 (16.18)	58.68 (17.17)	56.06 (17.76)	53.14 (31.87)	<.001
LDL-C^g^ (mg/dL), mean (SD)	97.55 (28.41)	104.67 (31.25)	108.84 (33.18)	107.78 (42.38)	<.001
eGFR^h^ (mL/min/1.73 m^2^), mean (SD)	98.03 (55.31)	96.4 (50.47)	95.26 (46.46)	94.67 (44.64)	<.001
Triglyceride (mg/dL), median (IQR)	51.45 (51.43-51.47)	79.15 (79.12-79.18)	111.27 (111.22-111.32)	194.1 (193.96-194.24)	<.001

a TyG: triglyceride-glucose.

b Not available.

c CKD: chronic kidney disease.

d SBP: systolic blood pressure.

e DBP: diastolic blood pressure.

f HDL-C: high-density lipoprotein-cholesterol.

g LDL-C: low-density lipoprotein-cholesterol.

h eGFR: estimated glomerular filtration rate.

### Incidence Rates, Absolute Incidence Difference, Cumulative Incidence, and HRs of ESKD by TyG Index, Fasting Glucose, and Triglyceride Quartiles

**Figure 1.**
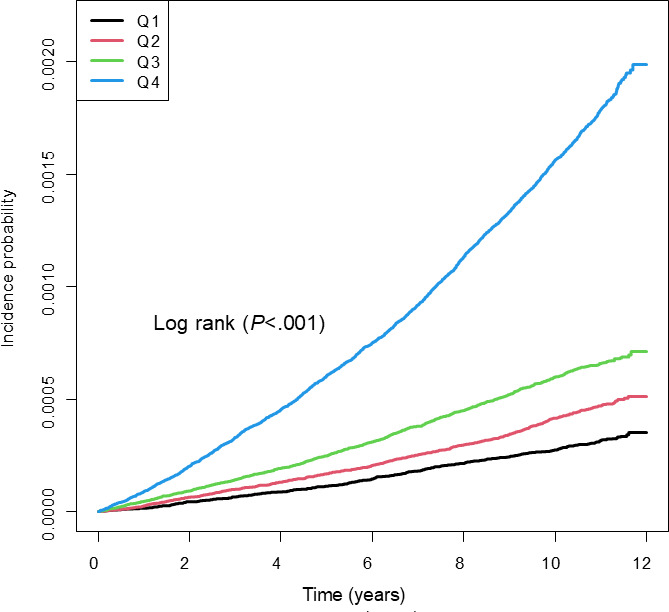
Cumulative incidence of end-stage kidney disease by the triglyceride-glucose (TyG) index quartiles: Kaplan-Meier analysis. Q: quartile.

The incidence rates, absolute incidence difference, 5- and 10-year cumulative incidence, and HRs of ESKD were analyzed across quartiles of the TyG index, fasting glucose, and triglycerides (Table 2). The incidence of ESKD increased progressively with higher TyG index quartiles (Figure 1). In both the unadjusted and adjusted models, the HRs of ESKD also increased significantly with increasing TyG index quartiles (all *P*<.001). In the fully adjusted Model 3, individuals in the highest TyG index quartile had an HR of 3.438 (95% CI 3.096‐3.818) compared with those in the lowest quartile.

Regarding fasting glucose, ESKD incidence rates and HRs were significantly higher in the highest quartile than in the lower quartiles (*P*<.001). The HR for ESKD in the highest fasting glucose quartile was 1.393 (95% CI 1.284‐1.511) compared with that in the lowest quartile in fully adjusted Model 3. However, the second (HR 0.836, 95% CI 0.761‐0.918) and third (HR 0.891, 95% CI 0.810‐0.979) quartiles showed significantly lower HRs compared to the lowest quartile group in adjusted Model 3.

For triglycerides, ESKD incidence rates and HRs showed patterns similar to those of the TyG index. The incidence rates increased progressively with the triglyceride quartiles. HRs for ESKD also increased significantly with increasing the triglyceride quartiles (*P*<.001). In the fully adjusted Model 3, the HR of the highest triglyceride quartile was 2.675 (95% CI 2.423‐2.953) compared to that of the lowest quartile. The second (HR 1.463, 95% CI 1.314‐1.628) and third (HR 1.693, 95% CI 1.527‐1.876) quartiles also showed significantly higher HRs than the lowest quartile in adjusted Model 3.

**Table 2.** Incidence rates, absolute incidence difference, cumulative incidence, and adjusted hazard ratios of end-stage kidney disease according to quartiles of the triglyceride-glucose index, fasting glucose, and triglyceride levels.

TyG^a^ index quartile	N	Event, n	Duration, PY^b^	IR^c^ per 1000 PY	Absolute incidence difference (per 1000 PY)	Cumulative incidence (%)		HR^d^ (95% CI)		
						5-year (95% CI)	10-year (95% CI)	Model 1^e^	Model 2^f^	Model 3^g^
Quartile 1	1,638,852	479	16,846,725.4	0.0284	0 (ref)	0.011 (0.010-0.013)	0.028 (0.025-0.030)	1 (ref)	1 (ref)	1 (ref)
Quartile 2	1,639,754	711	16,949,275.7	0.0420	0.013 (0.010 to 0.018)	0.017 (0.015-0.019)	0.042 (0.039-0.045)	1.472 (1.311-1.653)	1.425 (1.269-1.600)	1.400 (1.246-1.573)
Quartile 3	1,637,725	1014	16,976,870.8	0.0597	0.031 (0.027 to 0.036)	0.025 (0.023-0.028)	0.060 (0.056-0.064)	2.095 (1.879-2.335)	1.980 (1.775-2.209)	1.814 (1.624-2.026)
Quartile 4	1,639,532	2706	1,7002,806.5	0.1592	0.131 (0.124 to 0.137)	0.060 (0.057-0.064)	0.156 (0.150-0.162)	5.578 (5.062-6.148)	5.127 (4.646-5.658)	3.438 (3.096-3.818)
*P* value	—^h^	—	—	—	—	—	—	<.001	<.001	<.001
Fasting glucose										
Quartile 1	1,671,235	925	17,343,951.1	0.0533	0 (ref)	0.023 (0.021-0.026)	0.053 (0.049-0.056)	1 (ref)	1 (ref)	1 (ref)
Quartile 2	1,605,884	801	16,569,731.7	0.0483	−0.005 (−0.010 to 0.000)	0.021 (0.019-0.023)	0.049 (0.045-0.052)	0.908 (0.826-0.998)	0.883 (0.804-0.971)	0.891 (0.810-0.979)
Quartile 3	1,646,701	852	16,982,610.7	0.0502	−0.003 (−0.008 to 0.002)	0.022 (0.020-0.024)	0.050 (0.047-0.054)	0.943 (0.859-1.035)	0.886 (0.807-0.973)	0.836 (0.761-0.918)
Quartile 4	1,632,043	2332	16,879,384.8	0.1382	0.085 (0.078 to 0.091)	0.048 (0.045-0.052)	0.135 (0.129-0.141)	2.593 (2.403-2.799)	2.385 (2.209-2.576)	1.393 (1.284-1.511)
*P* value	—	—	—	—	—	—	—	<.001	<.001	<.001
Triglyceride										
Quartile 1	1,672,748	560	17,186,565.3	0.0326	0 (ref)	0.012 (0.011-0.014)	0.032 (0.029-0.035)	1 (ref)	1 (ref)	1 (ref)
Quartile 2	1,591,981	852	16,452,225.3	0.0518	0.019 (0.015 to 0.024)	0.019 (0.017-0.022)	0.051 (0.047-0.054)	1.586 (1.426-1.764)	1.521 (1.367-1.693)	1.463 (1.314-1.628)
Quartile 3	1,651,795	1154	17,122,569.9	0.0674	0.035 (0.030 to 0.040)	0.025 (0.023-0.028)	0.067 (0.063-0.072)	2.062 (1.864-2.281)	1.929 (1.743-2.135)	1.693 (1.527-1.876)
Quartile 4	1,639,339	2344	17,014,317.9	0.1378	0.105 (0.099 to 0.111)	0.057 (0.054-0.061)	0.136 (0.130-0.142)	4.212 (3.841-4.619)	3.830 (3.488-4.204)	2.675 (2.423-2.953)
*P* value	—	—	—	—	—	—	—	<.001	<.001	<.001

a TyG: triglyceride-glucose.

b PY: person-years.

c IR: incidence rate.

d HR: hazard ratio.

e Model 1: nonadjusted.

f Model 2: adjusted for age and sex.

g Model 3: adjusted for age, sex, BMI, smoking, drinking, regular exercise, diabetes medication, dyslipidemia medication, hypertension, low income, estimated glomerular filtration rate, and chronic kidney disease.

h Not available.

### Incidence Rates and HRs of ESKD by Deciles of the TyG Index, Fasting Glucose, and Triglycerides

ESKD incidence rates and HRs were assessed across deciles of the TyG index, fasting glucose, and triglyceride (Table 3). The incidence rates of ESKD increased progressively with increasing TyG index deciles. Similarly, HRs for ESKD showed a significant upward trend as TyG index deciles increased in both the unadjusted and adjusted models (all *P*<.001). In the fully adjusted Model 3, individuals in the highest TyG index decile had an HR of 5.944 (95% CI 5.022‐7.035) compared to those in the lowest decile.

Regarding fasting glucose, the incidence rates and HRs of ESKD were significantly higher in the highest decile than in the lower deciles (*P*<.001). In the fully adjusted Model 3, the HR for ESKD in the highest fasting glucose decile was 2.029 (95% CI 1.805‐2.282) compared to the lowest decile. However, the second through ninth deciles did not show statistically significant HRs compared to the lowest decile in the same model.

For triglycerides, ESKD incidence rates and HRs showed similar patterns to those of the TyG index. The incidence rates of ESKD increased progressively with increasing triglyceride deciles. Similarly, HRs for ESKD gradually increased with higher triglyceride deciles in both the unadjusted and adjusted models (all *P*<.001). In the fully adjusted Model 3, the HR for ESKD in the highest triglyceride decile was 4.042 (95% CI 3.433‐4.759) compared to that in the lowest triglyceride decile.

**Table 3.** Incidence rates and adjusted hazard ratios of end-stage kidney disease according to deciles of the triglyceride-glucose index, fasting glucose, and triglyceride levels.

TyG^a^ index decile	N	Event, n	Duration, PY^b^	IR^c^ per 1000 PY	HR^d^ (95% CI)		
					Model 1^e^	Model 2^f^	Model 3^g^
D1^h^	656,042	163	6,726,362.6	0.02	1 (ref)	1 (ref)	1 (ref)
D2	655,996	202	6,751,405.2	0.03	1.233 (1.003-1.515)	1.210 (0.984-1.487)	1.239 (1.008-1.524)
D3	655,258	235	6,757,963.2	0.03	1.432 (1.173-1.749)	1.390 (1.138-1.698)	1.425 (1.166-1.740)
D4	655,066	293	6,768,960.3	0.04	1.781 (1.471-2.157)	1.713 (1.414-2.075)	1.729 (1.427-2.095)
D5	656,244	297	6,791,309.8	0.04	1.799 (1.486-2.177)	1.716 (1.417-2.078)	1.715 (1.416-2.078)
D6	654,802	365	6,782,423.6	0.05	2.213 (1.839-2.661)	2.095 (1.741-2.521)	2.048 (1.700-2.466)
D7	655,955	395	6,801,963.6	0.06	2.387 (1.989-2.864)	2.242 (1.867-2.692)	2.135 (1.775-2.567)
D8	655,728	518	6,803,949.5	0.08	3.128 (2.623-3.730)	2.915 (2.443-3.479)	2.634 (2.203-3.149)
D9	655,160	652	6,797,631.0	0.10	3.941 (3.319-4.679)	3.639 (3.062-4.325)	3.038 (2.549-3.622)
D10	655,612	1790	6,793,709.5	0.26	10.827 (9.223-12.710)	9.850 (8.378-11.581)	5.944 (5.022-7.035)
*P* value	—^i^	—	—	—	<.001	<.001	<.001
Fasting glucose							
D1	676,873	395	7,052,603.2	0.06	1 (ref)	1 (ref)	1 (ref)
D2	604,458	337	6,263,814.7	0.05	0.963 (0.832-1.113)	0.971 (0.840-1.123)	1.014 (0.877-1.173)
D3	739,028	367	7,642,742.4	0.05	0.860 (0.746-0.991)	0.838 (0.727-0.966)	0.886 (0.769-1.022)
D4	621,214	331	6,413,596.6	0.05	0.925 (0.799-1.070)	0.883 (0.763-1.022)	0.923 (0.797-1.068)
D5	635,546	296	6,540,926.0	0.05	0.812 (0.698-0.944)	0.839 (0.722-0.976)	0.836 (0.718-0.972)
D6	646,017	320	6,659,217.4	0.05	0.861 (0.743-0.998)	0.817 (0.705-0.947)	0.816 (0.704-0.946)
D7	688,862	355	7,100,424.4	0.05	0.896 (0.777-1.034)	0.874 (0.757-1.009)	0.838 (0.725-0.967)
D8	658,165	382	6,796,143.4	0.06	1.007 (0.875-1.159)	0.943 (0.819-1.086)	0.866 (0.751-0.997)
D9	664,302	429	6,874,473.0	0.06	1.117 (0.974-1.281)	1.052 (0.918-1.207)	0.876 (0.763-1.006)
D10	621,398	1698	6,431,737.3	0.26	4.724 (4.234-5.270)	4.305 (3.855-4.808)	2.029 (1.805-2.282)
*P* value	—	—	—	—	<.001	<.001	<.001
Triglyceride							
D1	645,990	178	6,616,922.9	0.03	1 (ref)	1 (ref)	1 (ref)
D2	688,090	242	7,076,082.7	0.03	1.270 (1.046-1.541)	1.238 (1.020-1.502)	1.262 (1.040-1.531)
D3	638,511	266	6,586,656.6	0.04	1.497 (1.238-1.810)	1.440 (1.191-1.741)	1.440 (1.191-1.741)
D4	657,214	350	6,791,725.6	0.05	1.909 (1.593-2.286)	1.787 (1.492-2.141)	1.754 (1.464-2.102)
D5	634,924	376	6,567,402.8	0.06	2.120 (1.773-2.533)	1.996 (1.669-2.386)	1.918 (1.603-2.294)
D6	675,312	429	6,992,308.3	0.06	2.271 (1.907-2.704)	2.126 (1.784-2.532)	1.981 (1.661-2.362)
D7	647,027	457	6,711,946.2	0.07	2.518 (2.118-2.994)	2.294 (1.928-2.729)	2.034 (1.707-2.424)
D8	659,743	586	6,845,357.4	0.09	3.165 (2.677-3.743)	2.897 (2.448-3.428)	2.424 (2.044-2.874)
D9	653,633	713	6,784,455.6	0.11	3.886 (3.298-4.580)	3.490 (2.959-4.117)	2.732 (2.309-3.233)
D10	655,419	1313	6,802,820.2	0.19	7.138 (6.104-8.347)	6.331 (5.407-7.412)	4.042 (3.433-4.759)
*P* value	—	—	—	—	<.001	<.001	<.001

a TyG: triglyceride-glucose.

b PY: person-years.

c IR: incidence rate.

d HR: hazard ratio.

e Model 1: nonadjusted.

f Model 2: adjusted for age and sex.

g Model 3: adjusted for age, sex, BMI, smoking, drinking, regular exercise, diabetes medication, dyslipidemia medication, hypertension, low income, estimated glomerular filtration rate, and chronic kidney disease.

h D: decile.

i Not available.

### Subgroup Analyses of Incidence Rates and HRs of ESKD by TyG Index Quartiles

The incidence rates and adjusted hazard ratios (aHRs) of ESKD were evaluated across TyG index quartiles within various subgroups (Table 4). Interaction tests revealed that age, sex, obesity, MetS, diabetes, use of medications for diabetes or dyslipidemia, dyslipidemia, low income, eGFR, and CKD significantly influenced the association between the TyG index and ESKD (all *P* for interaction<.0001).

In subgroups stratified by age (20‐29 years and 30‐39 years), sex (male and female), obesity status (with or without), and CKD status (with or without), both the incidence rates and aHRs for ESKD significantly increased with higher TyG index quartiles. For participants without MetS, diabetes, or dyslipidemia and those not receiving medications for diabetes or dyslipidemia, the incidence rates and aHRs of ESKD also rose consistently across increasing TyG index quartiles.

In participants with dyslipidemia, the aHR for ESKD was significantly higher in the highest TyG index quartile than in the lowest quartile; however, the aHRs for the second and third quartiles were not significant. Among participants with diabetes, the aHRs were lower in the second and third quartiles, and the highest quartile did not show a significant difference compared with the first quartile. For participants with MetS and those receiving medications for diabetes or dyslipidemia, the aHRs for ESKD were not significantly different between the TyG index quartiles.

**Table 4.** Adjusted hazard ratios of end-stage kidney disease according to various subgroups. Adjusted for age, sex, BMI, smoking, drinking, regular exercise, diabetes medication, dyslipidemia medication, hypertension, low income, estimated glomerular filtration rate (eGFR), and chronic kidney disease (CKD).

TyG^a^ index	Number of patients	Event, n	Duration, PY^b^	IR^c^ per 1000 PY	HR^d^ (95% CI)	*P* for interaction
Age (years)						<.001
<30						
Quartile 1	894,184	214	9,102,734.5	0.0235	1 (ref)	
Quartile 2	743,906	238	7,604,879.7	0.0313	1.328 (1.104-1.597)	
Quartile 3	630,104	275	6,460,758.7	0.0426	1.763 (1.473-2.109)	
Quartile 4	487,580	701	5,011,557.2	0.1399	5.038 (4.311-5.888)	
≥30						
Quartile 1	744,668	265	7,743,990.9	0.0342	1 (ref)	
Quartile 2	895,848	473	9,344,396.1	0.0506	1.323 (1.157-1.513)	
Quartile 3	1,007,621	739	10,516,112.1	0.0703	1.562 (1.373-1.776)	
Quartile 4	1,151,952	2005	11,991,249.3	0.1672	2.703 (2.397-3.049)	
Sex						<.001
Male						
Quartile 1	975,747	366	10,059,535.3	0.0364	1 (ref)	
Quartile 2	975,000	529	10,135,210.0	0.0522	1.323 (1.157-1.513)	
Quartile 3	975,117	713	10,175,276.9	0.0701	1.562 (1.373-1.776)	
Quartile 4	975,291	1828	10,189,244.3	0.1794	2.703 (2.397-3.049)	
Female						
Quartile 1	663,105	113	6,787,190.0	0.0167	1 (ref)	
Quartile 2	664,754	182	6,814,065.7	0.0267	1.601 (1.266-2.025)	
Quartile 3	662,608	301	6,801,593.9	0.0443	2.582 (2.079-3.206)	
Quartile 4	664,241	878	6,813,562.2	0.1289	5.947 (4.876-7.253)	
Obesity						<.001
No						
Quartile 1	1,439,264	409	14,800,776.4	0.0276	1 (ref)	
Quartile 2	1,319,486	513	13,641,935.1	0.0376	1.292 (1.134-1.471)	
Quartile 3	1,164,343	662	12,070,099.3	0.0549	1.765 (1.559-1.998)	
Quartile 4	895,059	1326	9,288,242.4	0.1428	3.679 (3.284-4.123)	
Yes						
Quartile 1	199,588	70	2,045,949.0	0.0342	1 (ref)	
Quartile 2	320,268	198	3,307,340.7	0.0599	1.506 (1.147-1.978)	
Quartile 3	473,382	352	4,906,771.5	0.0717	1.508 (1.166-1.950)	
Quartile 4	744,473	1380	7,714,564.1	0.1789	2.425 (1.902-3.090)	
Metabolic syndrome						<.001
No						
Quartile 1	1,629,671	430	16,752,762.2	0.0257	1 (ref)	
Quartile 2	1,612,113	600	16,664,157.8	0.0360	1.411 (1.246-1.598)	
Quartile 3	1,513,148	707	15,683,481.0	0.0451	1.789 (1.585-2.019)	
Quartile 4	1,099,603	1008	11,397,312.7	0.0884	3.677 (3.274-4.129)	
Yes						
Quartile 1	9181	49	93,963.2	0.5215	1 (ref)	
Quartile 2	27,641	111	285,117.9	0.3893	0.920 (0.660-1.283)	
Quartile 3	124,577	307	1,293,389.8	0.2374	0.845 (0.627-1.139)	
Quartile 4	539,929	1698	5,605,493.8	0.3029	1.135 (0.856-1.505)	
Diabetes						<.001
No						
Quartile 1	1,635,767	435	16,814,934.1	0.0259	1 (ref)	
Quartile 2	1,631,845	649	16,867,366.5	0.0385	1.453 (1.287-1.640)	
Quartile 3	1,619,731	887	16,790,386.1	0.0528	1.914 (1.705-2.149)	
Quartile 4	1,541,397	1471	15,993,541.3	0.0920	3.020 (2.701-3.377)	
Yes						
Quartile 1	3085	44	31,791.2	1.3840	1 (ref)	
Quartile 2	7909	62	81,909.2	0.7569	0.650 (0.434-0.975)	
Quartile 3	17,994	127	186,484.7	0.6810	0.613 (0.427-0.880)	
Quartile 4	98,135	1235	1,009,265.2	1.2237	1.163 (0.843-1.605)	
Diabetes medication						<.001
No						
Quartile 1	1,637,356	438	16,831,389.4	0.0260	1 (ref)	
Quartile 2	1,637,462	657	16,925,781.9	0.0388	1.438 (1.274-1.622)	
Quartile 3	1,633,357	917	16,932,102.4	0.0542	1.892 (1.686-2.122)	
Quartile 4	1,618,700	2043	16,790,857.2	0.1217	3.759 (3.373-4.190)	
Yes						
Quartile 1	1496	41	15,336.0	2.6735	1 (ref)	
Quartile 2	2292	54	23,493.8	2.2985	0.692 (0.450-1.065)	
Quartile 3	4368	97	44,768.4	2.1667	0.617 (0.419-0.910)	
Quartile 4	20,832	663	211,949.3	3.1281	0.884 (0.631-1.240)	
Dyslipidemia						<.001
No						
Quartile 1	1,613,072	414	16,580,324.7	0.0250	1 (ref)	
Quartile 2	1,582,587	568	16,355,863.2	0.0347	1.384 (1.219-1.571)	
Quartile 3	1,528,478	728	15,840,855.1	0.0460	1.781 (1.577-2.012)	
Quartile 4	1,384,569	1476	14,358,412.0	0.1028	3.456 (3.084-3.874)	
Yes						
Quartile 1	25,780	65	266,400.6	0.2440	1 (ref)	
Quartile 2	57,167	143	593,412.5	0.2410	1.013 (0.753-1.363)	
Quartile 3	109,247	286	1,136,015.7	0.2518	1.021 (0.777-1.342)	
Quartile 4	254,963	1230	2,644,394.5	0.4651	1.486 (1.153-1.916)	
Dyslipidemia medication						<.001
No						
Quartile 1	1,613,072	414	16,580,324.7	0.0250	1 (ref)	
Quartile 2	1,582,587	568	16,355,863.2	0.0347	1.403 (1.241-1.587)	
Quartile 3	1,528,478	728	15,840,855.1	0.0460	1.798 (1.599-2.023)	
Quartile 4	1,384,569	1476	14,358,412.0	0.1028	3.992 (3.580-4.452)	
Yes						
Quartile 1	25,780	65	266,400.6	0.2440	1 (ref)	
Quartile 2	57,167	143	593,412.5	0.2410	0.926 (0.647-1.325)	
Quartile 3	109,247	286	1,136,015.7	0.2518	0.963 (0.695-1.335)	
Quartile 4	254,963	1230	2,644,394.5	0.4651	0.799 (0.589-1.084)	
CKD						<.001
No						
Quartile 1	1,595,127	333	16,368,449.1	0.0203	1 (ref)	
Quartile 2	1,597,840	453	16,493,096.1	0.0275	1.301 (1.129-1.500)	
Quartile 3	1,594,866	586	16,511,679.3	0.0355	1.564 (1.365-1.792)	
Quartile 4	1,591,942	1891	16,489,958.6	0.1147	3.703 (3.275-4.187)	
Yes						
Quartile 1	43,725	146	478,276.3	0.3053	1 (ref)	
Quartile 2	41,914	258	456,179.6	0.5656	1.721 (1.404-2.109)	
Quartile 3	42,859	428	465,191.5	0.9201	2.429 (2.010-2.935)	
Quartile 4	47,590	815	512,847.9	1.5892	2.909 (2.428-3.486)	

a TyG: triglyceride-glucose.

b PY: person-years.

c IR: incidence rate.

d HR: hazard ratio.

### Sensitivity Analysis of Incidence Rates and HRs of ESKD by Follow-Up and Time-Averaged TyG Index Quartiles

Sensitivity analysis was performed using TyG index quartiles at 2-year follow-up and time-averaged values from baseline and 2-year follow-up (Table 5). The incidence rates and aHRs of ESKD across TyG index quartiles at 2-year follow-up and for time-averaged values showed findings consistent with those of the baseline TyG index quartiles.

The incidence of ESKD increased progressively with higher TyG index quartiles at 2-year follow-up. In both the unadjusted and adjusted models, the HRs of ESKD also increased significantly with increasing TyG index quartiles (all *P*<.001). In the fully adjusted Model 3, individuals in the highest TyG index quartile had an HR of 2.860 (95% CI 2.466‐3.317) compared with those in the lowest quartile.

Regarding fasting glucose at 2-year follow-up, ESKD incidence rates and HRs were significantly higher in the highest quartile than in the lower quartiles (*P*<.001). The HR for ESKD in the highest fasting glucose quartile was 1.306 (95% CI 1.156‐1.475) compared to that in the lowest quartile in the fully adjusted Model 3. However, the second (HR 0.845, 95% CI 0.737‐0.973) and third (HR 0.838, 95% CI 0.729‐0.963) quartiles showed significantly lower HRs compared with the lowest quartile group in adjusted Model 3.

For triglycerides at 2-year follow-up, ESKD incidence rates and HRs showed patterns similar to those of the TyG index. The incidence rates increased progressively with the triglyceride quartiles. HRs for ESKD also increased significantly with increasing the triglyceride quartiles (*P*<.001). In the fully adjusted Model 3, the HR of the highest triglyceride quartile was 2.174 (95% CI 1.884‐2.508) compared to that of the lowest quartile. The second (HR 1.253, 95% CI 1.074‐1.461) and third (HR 1.445, 95% CI 1.246‐1.677) quartiles also showed significantly higher HRs than the lowest quartile in adjusted Model 3.

The incidence of ESKD increased progressively with higher time-averaged TyG index quartiles. In both the unadjusted and adjusted models, the HRs of ESKD also increased significantly with increasing TyG index quartiles (all *P*<.001). In the fully adjusted Model 3, individuals in the highest TyG index quartile had an HR of 3.449 (95% CI 2.937‐4.050) compared with those in the lowest quartile.

Regarding time-averaged fasting glucose, ESKD incidence rates and HRs were significantly higher in the highest quartile than in the lower quartiles (*P*<.001). The HR for ESKD in the highest fasting glucose quartile was 1.565 (95% CI 1.389‐1.763) compared to that in the lowest quartile in fully adjusted Model 3. However, the second (HR 0.906, 95% CI 0.788‐1.042) and third (HR 0.770, 95% CI 0.669‐0.886) quartiles showed significantly lower HRs compared with the lowest quartile group in adjusted Model 3.

For time-averaged triglycerides, ESKD incidence rates and HRs showed patterns similar to those of the TyG index. The incidence rates increased progressively with higher triglyceride quartiles. HRs for ESKD also increased significantly with increasing the triglyceride quartiles (*P*<.001). In fully adjusted Model 3, the HR of the highest triglyceride quartile was 2.092 (95% CI 1.810‐2.417) compared to that of the lowest quartile. The second (HR 1.320, 95% CI 1.131‐1.542) and third (HR 1.475, 95% CI 1.268‐1.715) quartiles also showed significantly higher HRs than the lowest quartile in adjusted Model 3.

**Table 5.** Incidence rates and adjusted hazard ratios of end-stage kidney disease according to quartiles of follow-up and time-averaged values of the triglyceride-glucose index, fasting glucose, and triglyceride levels.

2-year follow-up TyG^a^ index quartile	Number of patients	Event	Duration, PY^b^	IR^c^ per 1000 PY	HR^d^ (95% CI)		
					Model 1^e^	Model 2^f^	Model 3^g^
Quartile 1	966,738	247	8,117,937.8	0.03043	1 (ref)	1 (ref)	1 (ref)
Quartile 2	965,852	331	8,155,391.1	0.04059	1.332 (1.130-1.571)	1.288 (1.092-1.519)	1.188 (1.007-1.402)
Quartile 3	966,636	460	8,190,218.9	0.05616	1.842 (1.578-2.150)	1.737 (1.487-2.028)	1.428 (1.219-1.672)
Quartile 4	966,389	1145	8,190,761.2	0.13979	4.584 (3.995-5.260)	4.205 (3.661-4.831)	2.860 (2.466-3.317)
*P* value	—^h^	—	—	—	<.001	<.001	<.001
Fasting glucose							
Quartile 1	918,400	388	7,806,410.5	0.0497	1 (ref)	1 (ref)	1 (ref)
Quartile 2	983,768	389	8,315,230	0.04678	0.943 (0.819-1.085)	0.917 (0.797-1.056)	0.845 (0.734-0.973)
Quartile 3	952,913	419	8,028,657.7	0.05219	1.053 (0.917-1.209)	1.003 (0.874-1.152)	0.838 (0.729-0.963)
Quartile 4	1,010,534	987	8,504,010.9	0.11606	2.343 (2.083-2.635)	2.140 (1.901-2.409)	1.306 (1.156-1.475)
*P* value	—	—	—	—	<.001	<.001	<.001
Triglyceride							
Quartile 1	966,138	278	8,103,153.6	0.03431	1 (ref)	1 (ref)	1 (ref)
Quartile 2	976,553	402	8,243,730.1	0.04876	1.419 (1.218-1.654)	1.367 (1.173-1.593)	1.253 (1.074-1.461)
Quartile 3	953,353	527	8,078,334.6	0.06524	1.897 (1.640-2.193)	1.779 (1.538-2.058)	1.445 (1.246-1.677)
Quartile 4	969,571	976	8,229,090.8	0.1186	3.447 (3.017-3.938)	3.163 (2.766-3.617)	2.174 (1.884-2.508)
*P* value	—	—	—	—	<.001	<.001	<.001
Time-average of baseline and 2-year follow-up							
TyG index							
Quartile 1	966,403	205	8,094,556.1	0.02533	1 (ref)	1 (ref)	1 (ref)
Quartile 2	966,403	329	8,157,710.3	0.04033	1.589 (1.335-1.892)	1.536 (1.290-1.829)	1.432 (1.201-1.706)
Quartile 3	966,405	444	8,196,304.3	0.05417	2.133 (1.808-2.517)	2.011 (1.703-2.375)	1.685 (1.423-1.996)
Quartile 4	966,404	1205	8,205,738.4	0.14685	5.779 (4.983-6.701)	5.287 (4.550-6.143)	3.449 (2.937-4.050)
*P* value	—	—	—	—	<.001	<.001	<.001
Fasting glucose							
Quartile 1	982,056	404	8,324,979.1	0.04853	1 (ref)	1 (ref)	1 (ref)
Quartile 2	956,138	383	8,049,552.7	0.04758	0.983 (0.854-1.130)	0.955 (0.831-1.099)	0.906 (0.788-1.042)
Quartile 3	982,130	383	8,271,683.5	0.0463	0.956 (0.832-1.100)	0.900 (0.782-1.035)	0.770 (0.669-0.886)
Quartile 4	945,291	1013	8,008,093.7	0.1265	2.608 (2.324-2.927)	2.399 (2.136-2.696)	1.565 (1.389-1.763)
*P* value	—	—	—	—	<.001	<.001	<.001
Triglyceride							
Quartile 1	968,392	268	8,108,399.1	0.03305	1 (ref)	1 (ref)	1 (ref)
Quartile 2	975,884	407	8,232,289.3	0.04944	1.493 (1.280-1.742)	1.442 (1.236-1.683)	1.320 (1.131-1.542)
Quartile 3	953,357	511	8,082,020.2	0.06323	1.907 (1.645-2.211)	1.783 (1.537-2.068)	1.475 (1.268-1.715)
Quartile 4	967,982	997	8,231,600.4	0.12112	3.650 (3.189-4.177)	3.307 (2.885-3.791)	2.092 (1.810-2.417)
*P* value	—	—	—	—	<.001	<.001	<.001

a TyG: triglyceride-glucose.

b PY: person-years.

c IR: incidence rate.

d HR: hazard ratio.

e Model 1: nonadjusted.

f Model 2: adjusted for age and sex.

g Model 3: adjusted for age, sex, BMI, smoking, drinking, regular exercise, diabetes medication, dyslipidemia medication, hypertension, low income, estimated glomerular filtration rate, and chronic kidney disease.

h Not available.

## Discussion

### Principal Findings

This observational, population-based cohort study found that a higher TyG index was significantly associated with an increased risk of incident ESKD in young adults. This association was independent of traditional CKD risk factors and remained robust across subgroups based on age, sex, obesity, dyslipidemia, and CKD. These findings suggest that the TyG index, a marker of insulin resistance, may be useful for identifying young adults at risk of developing ESKD, and early intervention targeting insulin resistance may mitigate the pathogenesis of ESKD in young adults.

CKD is a leading global cause of mortality, affecting over 10% of the general population owing to the rising risk factors such as hypertension, diabetes, and obesity [1]. CKD progression to ESKD, which requires kidney replacement therapy, represents a significant global health issue. According to the International Society of Nephrology’s 2023 Global Kidney Health Atlas, the median incidence of treated ESKD was 146 individuals per million population in 84 countries, while the median prevalence of treated ESKD was 823 per million population in 95 countries [22]. Although CKD and ESKD are more prevalent in older adults, the global incidence of CKD is increasing in adolescents and young adults [3], who experience 30 times higher mortality rates than their healthy peers [23]. They face accumulating physiological complications and socioeconomic burdens from early life, predisposing them to poor long-term outcomes [7,24]. Therefore, identifying CKD risk factors early and implementing prompt interventions are critical for reducing the disease burden among young adults.

In this study, young adults with a higher TyG index were older, had a lower eGFR, and exhibited poorer metabolic health profiles than those with a lower TyG index. Moreover, they had higher BMI, waist circumference, blood pressure, and fasting plasma glucose, and poorer lipid profiles. Additionally, individuals with a higher TyG index showed higher rates of obesity, smoking, and alcohol consumption, as well as lower rates of regular exercise. They also had a greater prevalence of hypertension, diabetes, and dyslipidemia and were more likely to be on medications for diabetes or dyslipidemia than those with a lower TyG index. These findings align with previous studies [15,25,26]. During follow-up, ESKD incidence rates increased progressively with increasing TyG index, and the risk of ESKD significantly increased across both quartiles and deciles, independent of various ESKD risk factors. The individual contributions of the 2 TyG index components (ie, fasting plasma glucose and triglyceride levels) were separately investigated by analyzing the incidence rates and HRs across quartiles and deciles. Although fasting plasma glucose showed no significant effect, triglyceride levels demonstrated a weak effect in the decile-based analysis, which is consistent with a previous report [15]. Additionally, the impact of a high TyG index on ESKD pathogenesis was robust in subgroup analyses based on age, sex, obesity, dyslipidemia, and CKD. Sensitivity analyses using the 2-year follow-up TyG index and the time-averaged TyG index also showed significant associations with ESKD development. These findings suggest that the TyG index serves as more than a simple sum of its components (fasting plasma glucose and triglyceride levels) and has the potential to be a marker for assessing ESKD risk in young adults.

Previous studies have shown that the TyG index mediates the relationship between BMI and ESKD risk in middle-aged adults [26] and in the general population [15]. Among adolescents and young adults, higher SBP, fasting plasma glucose levels, and BMI have been identified as important factors contributing to CKD risk [3]. In this study, an elevated TyG index was associated with incident ESKD in young adults with both normal and high BMI, suggesting that a high TyG index, reflective of insulin resistance, plays a role in ESKD development both in connection with BMI and independently. Insulin resistance is known to cause glomerular hyperfiltration, sodium retention, tubular dysfunction, kidney inflammation, and fibrosis [11], all of which may contribute to ESKD progression through pathways independent of BMI. Additionally, previous research has linked the TyG index to the progression of coronary artery calcification in patients with CKD [27], suggesting that insulin resistance may further exacerbate ESKD by promoting atherosclerosis and vascular calcification. Insulin resistance contributes to atherogenesis and atherosclerosis progression by inducing macrophage apoptosis and causing damage to endothelial and vascular smooth muscle cells [28,29]. Hyperinsulinemia also promotes oxidative stress, structural and functional vessel injury [30], and osteogenic differentiation resulting in vascular calcification [31]. These findings highlight the importance of managing metabolic factors, in addition to weight reduction, to mitigate the detrimental effects of insulin resistance on progressive kidney injury.

Unexpectedly, the subgroup analysis showed that the TyG index was not associated with the risk of ESKD in young adults with diabetes or MetS, or those on medications for diabetes or dyslipidemia. Recent research has linked the TyG index to an increased risk of ESKD in patients with diabetes [16]. The reason for the discrepancy in our study is unclear. We speculate that there are several possible explanations. First, the population in this study differed from that in previous research, as this analysis focused exclusively on young adults aged 20‐39 years. Similarly, a recent study analyzing the National Health and Nutrition Examination Survey found no significant association between the TyG index and low eGFR (<60 mL/min/1.73 m^2^) in individuals aged 20‐40 years [25]. Second, among young adults with diabetes or those taking medications for diabetes, the association between the TyG index and ESKD may be weak. A previous study on CKD progression in type 2 diabetes suggested that the association between the TyG index and CKD was more pronounced in the early stages of the disease (eGFR >60 mL/min/1.73 m^2^) and in individuals without a history of oral diabetes medications [32]. Other studies have reported that while the TyG index correlates significantly with microalbuminuria, its association with CKD [33] or eGFR [34] is less evident. These findings suggest that the TyG index has a stronger influence in the early stages of CKD but a diminished effect in later stages, such as ESKD. Insulin resistance, which is characterized by hyperglycemia and dysfunctional lipid oxidation and usage, contributes to early glomerular damage in patients with diabetes. However, as dysglycemia progresses, multifactorial processes, including inflammation, oxidative stress, and metabolic acidosis, exacerbate kidney damage [14,33]. Third, among young adults with MetS or those taking medications for dyslipidemia, MetS and dyslipidemia status can change over time due to lifestyle modifications, medications, or illness [35], potentially weakening the association between the TyG index and ESKD risk during follow-up. Additionally, as the 2 components of the TyG index (ie, fasting glucose and triglyceride levels) overlap with the 5 diagnostic criteria for MetS, this may diminish the effect of the TyG index in patients with MetS. Fourth, in high-risk populations with established metabolic abnormalities (eg, diabetes, MetS, or abnormal glucose metabolism), the relationship between the TyG index and risk of CKD may be nonlinear or modified by concomitant therapies. A restricted cubic spline analysis from a cohort of hypertensive patients with abnormal glucose metabolism demonstrated a U-shaped association between the TyG index and incident CKD (eGFR <60 mL/min/1.73 m^2^ and/or proteinuria). The risk of CKD fell first and started to increase after the TyG index became higher than 8.94. Consistently, the risk of low eGFR also appeared to decrease first and then increase as the TyG quartile increased [36]. Taken together, these data partially support our finding that the TyG index predicted ESKD development mainly in metabolically healthier young adults, whereas its prognostic value was attenuated in those with preexisting diabetes or MetS, or those on medications for diabetes or dyslipidemia. Such patients have relatively higher baseline renal risk, and progression to ESKD is strongly influenced by disease duration, glycemic control, and renoprotective medications such as renin-angiotensin system inhibitors. Further studies are needed to elucidate the association between the TyG index and risk of ESKD in metabolically unhealthy young adults.

### Limitations

This study had several limitations. First, its observational design and limited scope of the database prevented the inclusion of various confounding factors that contribute to the pathogenesis of ESKD, such as inflammation, nutrition, comorbidities, other kidney diseases, and additional metabolic risk factors. Particularly, the lack of consideration of genetic or congenital kidney diseases is a limitation in this cohort of young adults. Second, as the TyG index does not adequately reflect changes in response to drug treatment or lifestyle modification, the evidence supporting its role in the development of ESKD remains limited. Third, the main analysis did not adjust for other metabolic risk factors due to the lack of such information in the NHIS database. Fourth, there is no biological validation supporting the association between the TyG index and ESKD. Finally, the insignificant association between the TyG index and ESKD in subgroup analyses of young adults with diabetes, MetS, and those taking medications for diabetes or dyslipidemia is also a limitation that needs further research. Despite these limitations, the findings of our study remain significant, as the study was based on a large population-based cohort with a longitudinal database. The study population uniquely focused on young adults, a group that has been underrepresented in ESKD research. Additionally, adjustments were made for established ESKD risk factors, eGFR, and income, and the TyG index was examined in quartiles and deciles, using follow-up and time-averaged values, enabling a comprehensive evaluation of its association with ESKD risk.

### Conclusions

In conclusion, this study demonstrated that a higher TyG index is significantly associated with an increased risk of incident ESKD in young adults. These findings suggest that the TyG index may have potential utility in identifying young adults at risk of developing ESKD. Further comprehensive studies are warranted to confirm the relationship and clarify the role of the TyG index in the development of ESKD in young adult populations.

## Abbreviations

aHR: adjusted hazard ratio
CKD: chronic kidney disease
DBP: diastolic blood pressure
eGFR: estimated glomerular filtration rate
ESKD: end-stage kidney disease
HDL-C: high-density lipoprotein-cholesterol
HR: hazard ratio
*ICD-10*: *International Statistical Classification of Diseases and Related Health Problems, 10th Revision*
MetS: metabolic syndrome
NHIS: National Health Insurance Service
SBP: systolic blood pressure
TyG: triglyceride-glucose
